# The quantum theory of light

**DOI:** 10.1098/rsta.2023.0349

**Published:** 2024-12-24

**Authors:** Stephen M. Barnett, John Jeffers

**Affiliations:** ^1^ School of Physics and Astronomy, University of Glasgow, Glasgow G12 8QQ, UK; ^2^ Department of Physics, University of Strathclyde, John Anderson Building, 107 Rottenrow, Glasgow G4 0NG, UK

**Keywords:** History of quantum theory, Photons, Non-clssical states of light

## Abstract

We present a brief introduction to the quantum theory of light as it is understood in the field of quantum optics. Our aim is not to review the topic, which would require a very extensive article (or even a book of several volumes) but rather to provide sufficient background to set the ideas in the following papers in their correct context.

This article is part of the theme issue ‘The quantum theory of light’.

## 1. Rodney Loudon and the quantum theory of light

The year 2023 marked 50 years since the publication of the first edition of Rodney Loudon’s classic and influential textbook, *The Quantum Theory of Light* [1]. Sadly, it was also the year following his death. The juxtaposition of these two events led us to suggest to *Philosophical Transactions A* a special issue in which invited authors might present at least part of the scope of modern quantum optics that Rodney and other pioneers have inspired. In this short article, we give the briefest of introductions to the field of quantum optics and how it has developed. Our aim is only to provide a setting for the papers that follow.

**Figure 1.**
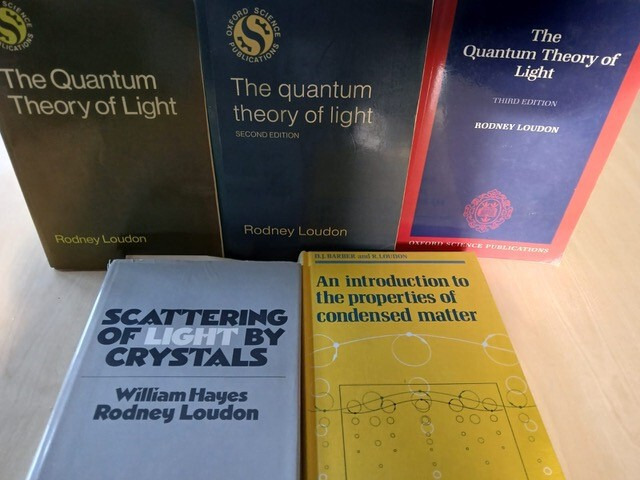
The three editions of *The Quantum Theory of Light* together with the two books authored by Rodney Loudon, with William Hayes and David Barber.

With the benefit of hindsight, we can see how the three editions of *The Quantum Theory of Light* [1–3], see figure 1 kept pace with the field of quantum optics and marked its advances.

## 2. Some history

When did the quantum theory of light begin? This is a question that may be answered in a number of ways depending on one’s perspective. Let us begin by examining some of the key events.

The theory of light and of its interaction with matter has been crucial to the development of quantum theory from the outset. It was the puzzling form of the black-body spectrum of light radiated by a hot body that first led Planck to his revolutionary quantum hypothesis [4–6] and this was followed by Einstein’s no less remarkable explanation for the photoelectric effect [7,8]. These two discoveries were central elements in the old quantum theory [9].

In 1926, Schrödinger introduced his famous equation for the wave function, marking the development of the new quantum theory. Incredibly, in this year, he also published a series of papers that covered, in essence, the entire student curriculum for future courses in quantum mechanics [10]. Following this, as early as 1927, Dirac introduced the quantum theory of light–matter interactions in which he gave a quantum theory of the electromagnetic field [11]. It is amusing to recall that in the introduction to his paper, Dirac wrote: ‘… hardly anything has been done up to the present on quantum electrodynamics.’ In fact, *nothing* had been done until then. There followed an explosion of interest in the new topic leading up to the work, in the late 1940s, of Tomonaga, Schwinger, Feynman and Dyson in which quantum electrodynamics emerged as the first fully functioning field theory [12].

Quantum optics might be said to have its origins in the genesis of the laser in the early 1960s, and by the early 1970s there was the need for books to lay out the foundations of the new discipline that had already introduced the quantum theory of coherence [13,14] and of photon counting [15]. There were just a few such books available at that time, those by Power [16], Louisell [17,18], Klauder and Sudarshan [19], Periňa [20] and, of course, by Loudon [1]. The situation at that time is summarized nicely in the opening paragraphs of the preface to Loudon’s work:

It used to be the case that all the basic quantum theory required for the explanation of experiments on light and its interaction with matter could be covered in a suitable chapter of a textbook on quantum mechanics. A knowledge of the theory of transition rates for absorption and emission of light by an atom sufficed for the understanding of most spectroscopic experiments. Books devoted to the theory of electromagnetic radiation and its interaction with matter, such as Heitler’s *Quantum theory of radiation*, were mainly concerned with photons in the high-energy region of the spectrum and the sophistications necessary to embrace relativistic corrections and to renormalize divergences.

The comparative simplicity of the theoretical tools required for the treatment of light in the visible region has been modified by the invention of the laser in 1960. The interpretation of experiments which use laser light has necessitated extensions of the previously developed theory in several directions.

Loudon’s text was remarkable in taking the reader from well-understood notions and ideas, familiar to advanced undergraduate physics students, through the challenges of the theory of elementary optical processes, optical coherence and, via the quantization of the radiation field, into photon optics and on to nonlinear optics.

By the time that the authors were introduced to the subject, quantum optics was becoming an established discipline with a rapidly growing international community and the needs of this community for an introductory text had changed. It was at this time that Loudon published the second edition of the book, *The Quantum Theory of Light* [2]. Superficially, the topics covered in the new edition were those from the first edition, but the range of material covered and its scope were greatly extended. The reason for this was the technological advances that made it possible to explore and demonstrate phenomena associated with the quantum theory of light in the laboratory. As Loudon explained in the preface to the second edition of *The Quantum Theory of Light* [2]:

The material is intended to bridge the gap between the more formal development of quantum electrodynamics and the application of the theory to the explanation of experimental results. The choice of theoretical topics is accordingly governed by the needs of experimental interpretation, but only a few representative experiments are discussed in any detail and the reader must look elsewhere for more complete accounts of the observational aspects.

**Figure 2.**
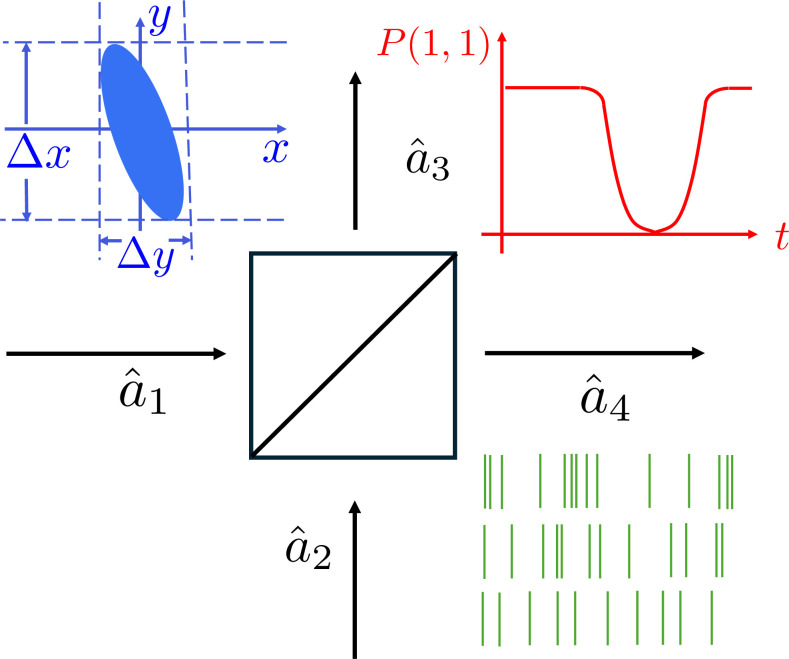
Schematic representation of three intrinsically quantum effects revealed in beam-splitting experiments: In blue (top left), we have the phase-space (Wigner function) representation of a squeezed state. In red (top right), we have the Hong–Ou–Mandel dip arising from two-photon interference. In green (bottom right), we have a representation of the time of detection of photocounts associated with bunched (chaotic) light, coherent laser light and finally non-classical anti-bunched light.

At this time, the cutting edge of quantum optics research was concerned with the so-called non-classical states of light. These are states that exhibit features with no classical explanation. More precisely, phenomena that cannot be explained by appealing to semiclassical theory in which the matter is treated quantum mechanically, but the electromagnetic field is governed by classical Maxwell theory. Important examples include squeezed states of light, with fluctuations or noise below that associated with electromagnetic vacuum [21], photon anti-bunching [22] and the wealth of phenomena appearing in the study of atoms confined to high-quality cavities [23]. A representation of some of these, particularly those demonstrated in optical interference, is given in figure 2.

By the turn of the millennium, quantum optics had advanced sufficiently that there was a need to update Loudon’s text and, in the year 2000, the third and final edition was published. It has the same gentle introduction and lucid explanations, but the range of topics was overhauled and modernized to fit the principles and techniques that had been developed over the preceding 20 years. There were, in particular, two striking changes, one obvious and the other rather subtle. The first was the inclusion of a very short introduction entitled ‘The Photon’. There he sought to caution the reader in both the use of the word ‘photon’ but, more significantly in their thinking about the quantum theory of light. It is clear that this section was a response (and, as ever, a highly pragmatic one) to a challenging and thought-provoking article by Lamb entitled ‘Anti-photon’ [24]. The second is the omission of a quotation present at the beginning of the second edition [25]: ‘We all *know* what light is: but it is not easy to *tell* what it is.’

We never thought to ask the reason for the omission. Perhaps Rodney, ever a modest man, felt that with the third edition, he really should be able to tell what light is. For us, and for many others, nobody ever told it better.

## 3. Special issue papers

Over the past 25 years, quantum optics has become a mature discipline. Many university physics departments have faculty researchers who are well-versed in the discipline and it is now common to find quantum optics lecture courses and laboratory work at the master’s and advanced undergraduate levels. There is also a somewhat bewildering range of texts to work or teach from, which contrasts strongly with the situation when Loudon wrote the first edition of *The Quantum Theory of Light* [1].

With maturity as a discipline has come great diversity and the insights and methods that quantum optics has produced have become part of other research topics. These include the cooling and trapping of atoms, ions and molecules, metrology and, perhaps most significantly, quantum information science. Accordingly, this special issue spans a broad range of applications, in line with the Loudon style of theory that could explain, predict the results of and inspire new experiments as well as provide insights into the mathematical structure.

The issue begins with a whimsically titled article on a quintessential quantum optical effect not always associated with Loudon. Nowadays two-photon interference is often styled the HOM effect, after Hong, Ou and Mandel, who made the first measurement of the classic dip in the coincidence rate of two detectors in 1987 (remarkably late—10 years after the first measurements of the less intuitive photon anti-bunching). Interference in optics normally requires coherence, which means it is typically demonstrated with light that emanates from the same original source. Loudon, working with Fearn on the theory at around the same time, pointed out one startling feature of two-photon interference, that the two photons can be, prior to their interaction at the beam splitter, entirely independent.

A series of articles follows on the interaction of light with matter, which of course changes and links the properties of both. Babiker’s article on quantum electrodynamics harks back to the origins of quantum optics and provides an analysis of the Röntgen term in the Hamiltonian and the Aharanov–Casher effects, both later measured. The next contribution provides an overview of the quantum theory of light in dielectric media. As late as the early 1990s, a fully canonical theory did not exist. Its successful development, linking absorption and dispersion, input and output fields, together with propagation and noise, paved the way for the resolution of a long-standing paradox on light momenta in media. The theme of momenta in media is continued by Baxter, who describes this paradox and its resolution, together with the photon drag effect, the deposition of optical momentum in a semiconductor that produces a current and led to the development of a type of photodetector.

Quantumness in light and its applications to quantum information are the theme of the next set of contributions, the first of which, a research article by Korolkova *et al.* proposes an operational distinction between quantum and classical optical entanglement, the former being based on statistics of two measurement outcomes. Optical angular momentum at the single-photon level is reviewed by Padgett. This property of light beams can be used as an alternative to the fundamental unit of quantum information, the qubit. Furthermore, it can be extended to higher-dimensional quantum information units, qunits (or qudits).

The next two papers develop this theme towards quantum information applications of light. The first, by Labay-Mora *et al.*, provides a review of neural networks and in particular concentrates on using squeezed states, with applications in quantum computing and memories. In a research article, Foulds *et al.* describe the detection of optical concentratable entanglement using controlled swap operations. They show a robustness to errors and suggest applications in quantum computing. The next contribution is a research article that first reviews retrodictive quantum mechanics in which the state of a quantum system prior to a measurement is written as the measured state. It applies this formalism to show results that suggest improvements in the distinction of optical states with an imperfect detector.

The review by Bordon and Vaccaro harks back to the first edition of the book that inspired this special issue, in which a form of quantum optical phase operator appeared. It led to the development of a phase formalism over around a decade, which resolved many seemingly intractable issues in the quantum theory of light. Gerry *et al.* return to the theme of quantum interference described in the opening article, this time describing effects associated with the coalescence of a single photon and a coherent state at a beam splitter and providing some statistical results that hark back to some old experiments. Twin optical beams, the measurement statistics of which can show entanglement, are a workhorse system in many optical quantum experiments. Gatti *et al.* model the production of such beams. Their theory is broad-ranging and can apply across different parameter regimes. Najafabadi *et al.* report research that links the two main approaches in quantum optics, which use, respectively, the Fock state basis and the continuous variable approach. They derive results using the Wigner function that applies to both photocounting experiments and homodyne detection and also provide experimental confirmation of their theory. A more recent theory is described in the research article by Croke *et al.* on using indefinite causal order as a measurement tool for quantum optics. One essential difference between quantum and classical physics is that in the former, order matters. This article shows how one might use the intrinsic indefiniteness of the order of operations in a Sagnac interferometer to measure the Pancharatnam–Berry phase. The next contribution, by Iqbal *et al.,* is a review of quantum metrology approaches in imaging resolution, suggesting that previously claimed advantages over classical physics may not be quite so straightforward.

The special issue is rounded off with two contributions, the first from nonlinear optics. Oppo and Firth review optical cavity solitons and their application in photonic devices, showing the universal applicability of the theory across multiple platforms. Last, but certainly not least, Herrera and Barnes use an open-system master-equation approach typical in quantum optics to model strongly coupled organic microcavities and the complexities that arise from such systems.

We are grateful to all the authors who have contributed to this special issue. They have covered a truly eclectic range of topics as befits the very versatile discipline into which quantum optics has grown.

The field is thriving and new and exciting developments may be confidently anticipated over the next 50 years.

## Data Availability

This article has no additional data.
